# Crystal structure of bis­(2,2′:6′,2′′-terpyridine-κ^3^ *N*,*N*′,*N*′′)nickel(II) dicyanidoaurate(I)

**DOI:** 10.1107/S1600536814024672

**Published:** 2014-11-19

**Authors:** Frankie White, Richard E. Sykora

**Affiliations:** a University of South Alabama, Department of Chemistry, Mobile, AL 36688, USA

**Keywords:** crystal structure, terpyridine, aurophilic inter­action, dicyanidoaurate

## Abstract

The title compound, [Ni(C_15_H_11_N_3_)_2_][Au(CN)_2_]_2_, is an ionic compound composed of bis­(2,2′:6′,2′′-terpyridine)­nickel(II) dications and dicyanidoaurate(I) anions in a 1:2 ratio. The two tridentate terpyridine ligands define the coordination of the Ni^2+^ cation, resulting in a nearly octa­hedral coordination sphere, although there is not any imposed crystallographic symmetry about the Ni^2+^ site. The two nearly linear dicyanidoaurate(I) anions [C—Au—C = 179.0 (2) and 178.2 (2)°] contain a short aurophilic inter­action of 3.1017 (3) Å. The structure does not demonstrate any π–π stacking. Non-classical C—H⋯N inter­actions between the cations and anions build up a three-dimensional network.

## Abstract 2

In an ionic compound composed of bis­(2,2′:6′,2′′-terpyridine)­nickel(II) dications and dicyanidoaurate(I) dianions in a 1:2 ratio, the two tridentate terpyridine ligands define the coordination of the Ni^2+^ cation, resulting in a nearly octa­hedral coordination sphere, although there is not any imposed crystallographic symmetry about the Ni^2+^ site.

## Chemical context   

Derivatives of the compound [*M*(terpy)_2_](*X*) (*M* = transition metal; terpy = 2,2′:6′,2′′-terpyridine; *X* = anion) have been known since the 1970′s (Harris & Lockyer, 1970 ▶). Transition metal–terpyridine complexes have been known to exhibit inter­esting properties such as their photophysical and spin-state properties (Pal *et al.*, 2014 ▶). These allow transition metal–terpyridine complexes to have useful applications in mol­ecular electronics and as building blocks for copolymers (Katz *et al.*, 2008 ▶; Pal *et al.*, 2014 ▶; Schubert *et al.*, 2001 ▶). However, it was not until recently that the incorporation of gold cyanidometallates has been introduced into these systems (Ovens *et al.*, 2010 ▶). We report here the synthesis and crystal structure of another metal–terpyridine cyanidoaurate, [Ni(C_15_H_11_N_3_)_2_][Au(CN)_2_]_2_, (I).
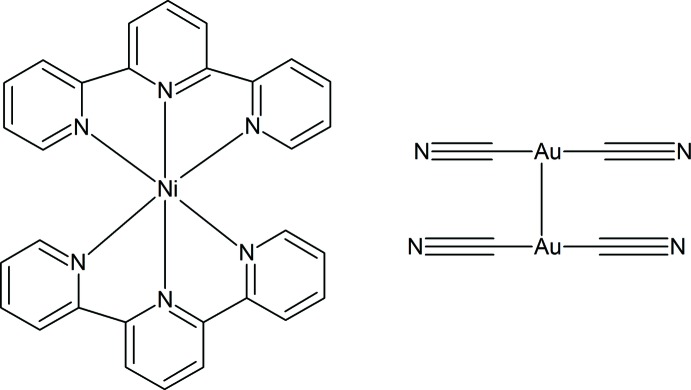


## Structural commentary   

**Figure 1:**
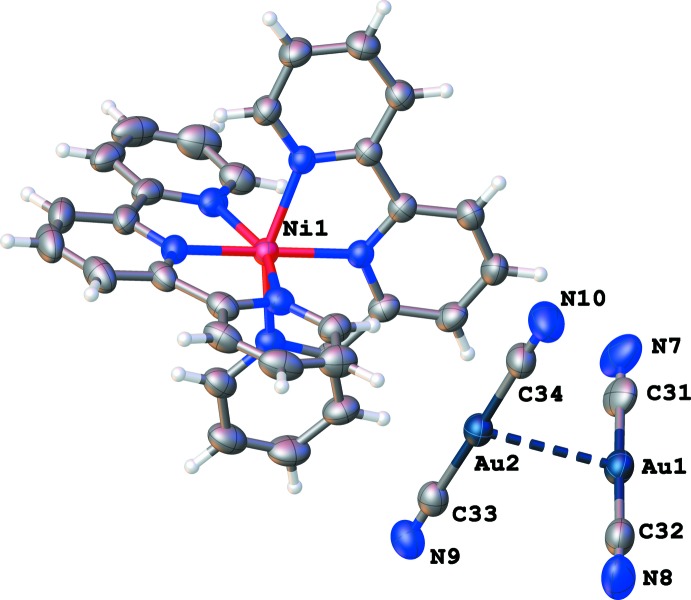
The mol­ecular structure of (I), with the atom-numbering scheme. Displacement ellipsoids for non-hydrogen atoms are drawn at the 50% probability level.

The structure of compound (I) contains an Ni^2+^ ion coordin­ated by two tridentate 2,2′:6′,2"-terpyridine ligands. The coordination of the terpyridine ligands around the metal cation gives an approximate octa­hedral coordination sphere. Included in the structure are two dicyanidoaurate(I) anions that are non-coordinating to the Ni^2+^ cation, as shown in Fig. 1 ▶. Recently, the compound [Ni(terpy)][Au(Br)_2_(CN)_2_]_2_ was synthesized and analysed (Ovens *et al.*, 2010 ▶). Its crystal structure contains a gold(III) cyanidometallate anion and a complex [Ni(terpy)]^2+^ cation. The title compound has some similarity, given that it too contains a [Ni(terpy)]^2+^ cation with dicyanidoaurate(I) anions. However, the important difference between the two compounds is that there are no metal–metal inter­actions in the [Ni(terpy)][Au(Br)_2_(CN)_2_]_2_ structure containing the *d* ^8^ Au(III) ion, whereas the [Ni(terpy)_2_][Au(CN)_2_]_2_ structure contains a *d* ^10^ gold(I) dicyanidoaurate(I) anion that has a strong propensity to form aurophilic inter­actions. This makes the title compound of inter­est because it contains short aurophilic inter­actions, contained within dimeric [Au(CN)_2_]_2_ moieties, with Au⋯Au distances of 3.1017 (3) Å (Fig. 1 ▶).

## Supra­molecular features   

**Figure 2:**
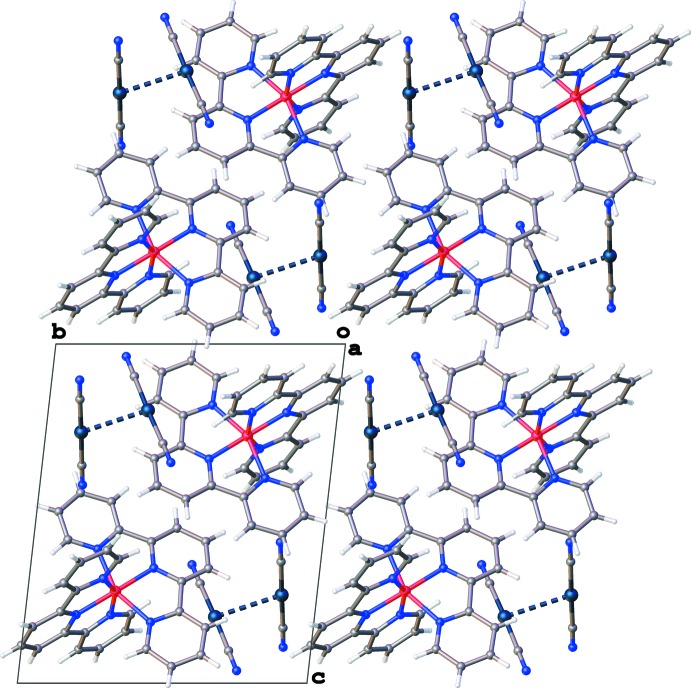
An illustration of the packing of the mol­ecular entities of (I).

A packing diagram of the title compound is illustrated in Fig. 2 ▶. There are not any classical hydrogen bonds within the structure of the title compound. However, the cation and anion are stabilized by relatively weak non-classical hydrogen-bonding inter­actions from H atoms on the terpyridine rings to terminal N atoms on the cyanidometallates. There are six such inter­actions ranging from 3.235 (7) to 3.421 (7) Å, if using a *D⋯A* distance of 3.5 Å as the upper defined limit. Details of the inter­actions can be found in Table 1 ▶. The other type of non-classical inter­molecular inter­actions that exists in the structure is the one aurophilic inter­action discussed in the *Structural commentary* above. There are no π–π stacking inter­actions in the structure.

## Synthesis and crystallization   

Ethanol solutions of 0.1 *M* Ni(NO_3_)_2_ (1 ml) and 0.1 *M* 2,2′:6′.2"-terpyridine (1 ml) were mixed together. Following the mixture of these two compounds, 2 ml of 0.05 *M* KAu(CN)_2_ (50:50 ethanol/water *v*/*v*) was added dropwise. A precipitate formed and the suspension was mixed thoroughly and centrifuged. The brownish-red solution was deca­nted from the solid precipitate and placed in a test tube to allow for slow evaporation. After approximately one week, the formation of brownish-red crystals had begun. The grown single crystals were then gathered and isolated.

## Refinement   

Crystal data, data collection and structure refinement details are summarized in Table 2 ▶. H atoms were placed in calculated positions and allowed to ride on their parent atoms during subsequent refinement, with *U*_iso_(H) = 1.2*U*_eq_(C) and C—H distances of 0.93 Å.

## Supplementary Material

Crystal structure: contains datablock(s) I, New_Global_Publ_Block. DOI: 10.1107/S1600536814024672/wm5063sup1.cif

Structure factors: contains datablock(s) I. DOI: 10.1107/S1600536814024672/wm5063Isup2.hkl

CCDC reference: 1033527

Additional supporting information:  crystallographic information; 3D view; checkCIF report

## Figures and Tables

**Table 1:** Hydrogen-bond geometry (, )

*D*H*A*	*D*H	H*A*	*D* *A*	*D*H*A*
C1H1N10^i^	0.93	2.57	3.235(7)	129
C7H7N8^ii^	0.93	2.51	3.356(9)	151
C8H8N9^iii^	0.93	2.54	3.421(7)	157
C22H22N10^iv^	0.93	2.43	3.364(8)	179
C23H23N7	0.93	2.51	3.274(7)	139
C30H30N9^v^	0.93	2.41	3.280(7)	156

Symmetry codes: (i) 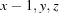; (ii) 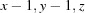; (iii) 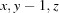; (iv) 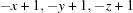; (v) 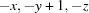.

**Table 2:** Experimental details

Crystal data	
Chemical formula	[Ni(C_15_H_11_N_3_)_2_][Au(CN)_2_]_2_
*M*_r_	1023.26
Crystal system, space group	Triclinic, *P* 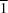
Temperature (K)	180
*a*, *b*, *c* ()	8.8374(3), 12.6707(4), 14.7497(4)
, , ()	83.401(2), 88.788(3), 81.078(3)
*V* (^3^)	1620.82(9)
*Z*	2
Radiation type	Mo *K*
(mm^1^)	9.65
Crystal size (mm)	0.09 0.06 0.05

Data collection	
Diffractometer	Agilent Xcalibur Eos
Absorption correction	Multi-scan (*CrysAlis PRO*; Agilent, 2014 ▶)
*T*_min_, *T*_max_	0.299, 1.000
No. of measured, independent and observed [*I* > 2(*I*)] reflections	41260, 5928, 5305
*R*_int_	0.045
(sin /)_max_ (^1^)	0.602

Refinement	
*R*[*F* ^2^ > 2(*F* ^2^)], *wR*(*F* ^2^), *S*	0.027, 0.070, 1.06
No. of reflections	5928
No. of parameters	424
H-atom treatment	H-atom parameters constrained
_max_, _min_ (e ^3^)	1.18, 0.51

Computer programs: *CrysAlis PRO* (Agilent, 2014 ▶), *SHELXS97* and *SHELXL97* (Sheldrick, 2008 ▶), *OLEX2* (Dolomanov *et al.*, 2009 ▶) and *publCIF* (Westrip, 2010 ▶).

## supplementary crystallographic information

### Crystal data

**Table:**

[Ni(C_15_H_11_N_3_)_2_][Au(CN)_2_]_2_	*Z* = 2
*M**_r_* = 1023.26	*F*(000) = 964
Triclinic, *P*1	*D*_x_ = 2.097 Mg m^−^^3^
*a* = 8.8374 (3) Å	Mo *K*α radiation, λ = 0.7107 Å
*b* = 12.6707 (4) Å	Cell parameters from 15517 reflections
*c* = 14.7497 (4) Å	θ = 2.6–28.0°
α = 83.401 (2)°	µ = 9.65 mm^−^^1^
β = 88.788 (3)°	*T* = 180 K
γ = 81.078 (3)°	Irregular, red
*V* = 1620.82 (9) Å^3^	0.09 × 0.06 × 0.05 mm

### Data collection

**Table:**

Agilent Xcalibur Eos diffractometer	5928 independent reflections
Radiation source: Enhance (Mo) X-ray Source	5305 reflections with *I* > 2σ(*I*)
Graphite monochromator	*R*_int_ = 0.045
Detector resolution: 16.0514 pixels mm^-1^	θ_max_ = 25.4°, θ_min_ = 2.6°
ω scans	*h* = −10→10
Absorption correction: multi-scan (*CrysAlis PRO*; Agilent, 2014)	*k* = −15→15
*T*_min_ = 0.299, *T*_max_ = 1.000	*l* = −17→17
41260 measured reflections	

### Refinement

**Table:**

Refinement on *F*^2^	Primary atom site location: structure-invariant direct methods
Least-squares matrix: full	Secondary atom site location: difference Fourier map
*R*[*F*^2^ > 2σ(*F*^2^)] = 0.027	Hydrogen site location: inferred from neighbouring sites
*wR*(*F*^2^) = 0.070	H-atom parameters constrained
*S* = 1.06	*w* = 1/[σ^2^(*F*_o_^2^) + (0.0357*P*)^2^ + 1.8409*P*] where *P* = (*F*_o_^2^ + 2*F*_c_^2^)/3
5928 reflections	(Δ/σ)_max_ = 0.001
424 parameters	Δρ_max_ = 1.18 e Å^−^^3^
0 restraints	Δρ_min_ = −0.51 e Å^−^^3^

### Special details

**Table:**

Geometry. All e.s.d.'s (except the e.s.d. in the dihedral angle between two l.s. planes) are estimated using the full covariance matrix. The cell e.s.d.'s are taken into account individually in the estimation of e.s.d.'s in distances, angles and torsion angles; correlations between e.s.d.'s in cell parameters are only used when they are defined by crystal symmetry. An approximate (isotropic) treatment of cell e.s.d.'s is used for estimating e.s.d.'s involving l.s. planes.
Refinement. Refinement of *F*^2^ against ALL reflections. The weighted *R*-factor *wR* and goodness of fit *S* are based on *F*^2^, conventional *R*-factors *R* are based on *F*, with *F* set to zero for negative *F*^2^. The threshold expression of *F*^2^ > 2σ(*F*^2^) is used only for calculating *R*-factors(gt) *etc*. and is not relevant to the choice of reflections for refinement. *R*-factors based on *F*^2^ are statistically about twice as large as those based on *F*, and *R*- factors based on ALL data will be even larger.

### Fractional atomic coordinates and isotropic or equivalent isotropic displacement parameters (Å^2^)

**Table:**

	*x*	*y*	*z*	*U*_iso_*/*U*_eq_	
Au1	0.39267 (2)	0.878031 (15)	0.259898 (14)	0.05010 (8)	
Au2	0.37167 (2)	0.656248 (15)	0.195383 (13)	0.04589 (7)	
Ni1	0.03978 (6)	0.30283 (4)	0.27059 (4)	0.03278 (13)	
N4	0.1366 (4)	0.2245 (3)	0.3954 (2)	0.0343 (8)	
N5	0.0613 (4)	0.4262 (3)	0.3394 (2)	0.0341 (8)	
N6	−0.0595 (4)	0.4355 (3)	0.1801 (2)	0.0367 (8)	
N3	0.2540 (4)	0.2959 (3)	0.2017 (2)	0.0386 (8)	
N2	0.0398 (4)	0.1761 (3)	0.2020 (2)	0.0371 (8)	
N1	−0.1736 (4)	0.2555 (3)	0.3086 (3)	0.0407 (9)	
C21	0.1440 (5)	0.4070 (4)	0.4172 (3)	0.0355 (9)	
C20	0.1790 (5)	0.2913 (3)	0.4515 (3)	0.0343 (9)	
C26	−0.0609 (5)	0.5321 (4)	0.2110 (3)	0.0385 (10)	
C16	0.1583 (5)	0.1185 (4)	0.4230 (3)	0.0412 (10)	
H16	0.1277	0.0722	0.3847	0.049*	
C19	0.2464 (5)	0.2534 (4)	0.5352 (3)	0.0428 (11)	
H19	0.2749	0.3008	0.5731	0.051*	
C22	0.1874 (6)	0.4908 (4)	0.4572 (3)	0.0430 (11)	
H22	0.2452	0.4773	0.5104	0.052*	
C30	−0.1275 (5)	0.4324 (4)	0.1000 (3)	0.0458 (11)	
H30	−0.1253	0.3665	0.0777	0.055*	
C5	−0.1995 (5)	0.1651 (4)	0.2730 (3)	0.0436 (11)	
C11	0.2842 (5)	0.2140 (4)	0.1487 (3)	0.0405 (10)	
C24	0.0583 (6)	0.6150 (4)	0.3379 (3)	0.0446 (11)	
H24	0.0272	0.6849	0.3110	0.054*	
C23	0.1440 (6)	0.5948 (4)	0.4175 (3)	0.0452 (11)	
H23	0.1723	0.6517	0.4442	0.054*	
C15	0.3549 (5)	0.3636 (4)	0.2029 (3)	0.0437 (11)	
H15	0.3334	0.4202	0.2384	0.052*	
C25	0.0205 (5)	0.5273 (3)	0.2995 (3)	0.0367 (10)	
C1	−0.2761 (6)	0.3004 (4)	0.3657 (3)	0.0491 (12)	
H1	−0.2587	0.3625	0.3892	0.059*	
C10	0.1623 (6)	0.1451 (4)	0.1495 (3)	0.0407 (11)	
C18	0.2705 (6)	0.1439 (4)	0.5616 (3)	0.0503 (12)	
H18	0.3180	0.1167	0.6169	0.060*	
C6	−0.0791 (6)	0.1213 (4)	0.2111 (3)	0.0434 (11)	
C17	0.2241 (6)	0.0754 (4)	0.5057 (3)	0.0480 (12)	
H17	0.2368	0.0017	0.5233	0.058*	
C27	−0.1337 (6)	0.6270 (4)	0.1636 (4)	0.0503 (12)	
H27	−0.1335	0.6924	0.1863	0.060*	
C9	0.1660 (7)	0.0561 (4)	0.1017 (4)	0.0540 (14)	
H9	0.2483	0.0354	0.0640	0.065*	
C14	0.4912 (6)	0.3535 (5)	0.1532 (4)	0.0527 (13)	
H14	0.5592	0.4023	0.1550	0.063*	
C4	−0.3309 (6)	0.1213 (4)	0.2956 (4)	0.0551 (14)	
H4	−0.3497	0.0606	0.2704	0.066*	
C12	0.4186 (6)	0.1984 (5)	0.0993 (3)	0.0509 (13)	
H12	0.4394	0.1407	0.0649	0.061*	
C13	0.5218 (6)	0.2694 (5)	0.1016 (4)	0.0576 (15)	
H13	0.6123	0.2602	0.0680	0.069*	
C7	−0.0793 (7)	0.0300 (4)	0.1661 (4)	0.0546 (14)	
H7	−0.1607	−0.0088	0.1728	0.066*	
C3	−0.4349 (6)	0.1692 (6)	0.3568 (4)	0.0683 (18)	
H3	−0.5229	0.1397	0.3734	0.082*	
C32	0.5489 (7)	0.8954 (4)	0.1631 (4)	0.0557 (14)	
C8	0.0441 (8)	−0.0008 (4)	0.1118 (4)	0.0609 (16)	
H8	0.0457	−0.0611	0.0812	0.073*	
C2	−0.4084 (6)	0.2576 (6)	0.3916 (4)	0.0638 (16)	
H2	−0.4773	0.2899	0.4324	0.077*	
C31	0.2372 (8)	0.8628 (4)	0.3579 (4)	0.0621 (15)	
N8	0.6378 (7)	0.9036 (5)	0.1061 (4)	0.0765 (16)	
C29	−0.2014 (6)	0.5252 (5)	0.0493 (4)	0.0579 (14)	
H29	−0.2469	0.5213	−0.0062	0.070*	
C28	−0.2056 (7)	0.6228 (5)	0.0831 (4)	0.0615 (15)	
H28	−0.2570	0.6853	0.0514	0.074*	
N7	0.1488 (8)	0.8529 (4)	0.4154 (4)	0.0830 (18)	
N9	0.1436 (6)	0.7521 (4)	0.0368 (3)	0.0576 (11)	
C33	0.2250 (6)	0.7165 (4)	0.0954 (4)	0.0477 (12)	
N10	0.6060 (7)	0.5566 (4)	0.3494 (4)	0.0765 (16)	
C34	0.5206 (7)	0.5917 (4)	0.2936 (4)	0.0536 (13)	

### Atomic displacement parameters (Å^2^)

**Table:**

	*U*^11^	*U*^22^	*U*^33^	*U*^12^	*U*^13^	*U*^23^
Au1	0.05730 (14)	0.03953 (12)	0.05524 (14)	−0.00962 (9)	−0.00594 (10)	−0.00889 (9)
Au2	0.05153 (13)	0.04074 (12)	0.04712 (12)	−0.00948 (9)	−0.00261 (9)	−0.00856 (8)
Ni1	0.0342 (3)	0.0307 (3)	0.0338 (3)	−0.0050 (2)	−0.0024 (2)	−0.0048 (2)
N4	0.0348 (19)	0.0319 (19)	0.0361 (19)	−0.0043 (15)	−0.0009 (15)	−0.0048 (15)
N5	0.0363 (19)	0.0318 (19)	0.0347 (19)	−0.0057 (15)	0.0012 (15)	−0.0052 (15)
N6	0.0339 (19)	0.040 (2)	0.0350 (19)	−0.0041 (16)	−0.0002 (15)	−0.0017 (16)
N3	0.039 (2)	0.040 (2)	0.035 (2)	−0.0022 (17)	−0.0027 (16)	−0.0018 (16)
N2	0.042 (2)	0.0314 (19)	0.038 (2)	−0.0051 (16)	−0.0059 (17)	−0.0048 (16)
N1	0.037 (2)	0.043 (2)	0.041 (2)	−0.0082 (17)	−0.0077 (17)	0.0007 (17)
C21	0.033 (2)	0.034 (2)	0.039 (2)	−0.0037 (18)	0.0030 (18)	−0.0053 (18)
C20	0.033 (2)	0.035 (2)	0.034 (2)	−0.0026 (18)	0.0008 (18)	−0.0054 (18)
C26	0.038 (2)	0.034 (2)	0.042 (2)	−0.0034 (19)	0.0026 (19)	−0.0027 (19)
C16	0.046 (3)	0.034 (2)	0.044 (3)	−0.007 (2)	−0.003 (2)	−0.0037 (19)
C19	0.046 (3)	0.043 (3)	0.040 (3)	−0.007 (2)	−0.006 (2)	−0.005 (2)
C22	0.051 (3)	0.043 (3)	0.037 (2)	−0.010 (2)	0.000 (2)	−0.009 (2)
C30	0.042 (3)	0.057 (3)	0.038 (3)	−0.008 (2)	−0.005 (2)	−0.001 (2)
C5	0.043 (3)	0.044 (3)	0.043 (3)	−0.012 (2)	−0.011 (2)	0.010 (2)
C11	0.041 (2)	0.044 (3)	0.033 (2)	0.005 (2)	−0.0050 (19)	−0.0035 (19)
C24	0.055 (3)	0.030 (2)	0.047 (3)	−0.003 (2)	0.005 (2)	−0.003 (2)
C23	0.058 (3)	0.037 (3)	0.044 (3)	−0.013 (2)	0.001 (2)	−0.010 (2)
C15	0.042 (3)	0.041 (3)	0.047 (3)	−0.005 (2)	−0.002 (2)	0.001 (2)
C25	0.035 (2)	0.031 (2)	0.042 (2)	0.0004 (18)	0.0039 (19)	−0.0009 (18)
C1	0.044 (3)	0.057 (3)	0.043 (3)	0.000 (2)	−0.003 (2)	−0.002 (2)
C10	0.049 (3)	0.038 (2)	0.033 (2)	0.005 (2)	−0.010 (2)	−0.0073 (19)
C18	0.057 (3)	0.049 (3)	0.042 (3)	−0.006 (2)	−0.009 (2)	0.004 (2)
C6	0.051 (3)	0.037 (2)	0.043 (3)	−0.011 (2)	−0.016 (2)	0.000 (2)
C17	0.054 (3)	0.036 (2)	0.051 (3)	−0.005 (2)	−0.007 (2)	0.004 (2)
C27	0.053 (3)	0.039 (3)	0.054 (3)	−0.002 (2)	−0.003 (2)	0.008 (2)
C9	0.066 (3)	0.050 (3)	0.044 (3)	0.010 (3)	−0.012 (2)	−0.016 (2)
C14	0.038 (3)	0.061 (3)	0.056 (3)	−0.006 (2)	0.002 (2)	0.005 (3)
C4	0.053 (3)	0.050 (3)	0.062 (3)	−0.020 (3)	−0.020 (3)	0.017 (3)
C12	0.049 (3)	0.063 (3)	0.035 (3)	0.007 (3)	0.001 (2)	−0.004 (2)
C13	0.038 (3)	0.082 (4)	0.045 (3)	0.001 (3)	0.004 (2)	0.009 (3)
C7	0.067 (4)	0.041 (3)	0.059 (3)	−0.015 (3)	−0.023 (3)	−0.006 (2)
C3	0.040 (3)	0.089 (5)	0.070 (4)	−0.022 (3)	−0.009 (3)	0.032 (4)
C32	0.062 (4)	0.049 (3)	0.061 (4)	−0.016 (3)	−0.008 (3)	−0.013 (3)
C8	0.083 (4)	0.041 (3)	0.059 (3)	−0.002 (3)	−0.025 (3)	−0.015 (3)
C2	0.039 (3)	0.086 (5)	0.060 (4)	−0.004 (3)	0.002 (3)	0.008 (3)
C31	0.083 (4)	0.032 (3)	0.072 (4)	−0.003 (3)	0.005 (3)	−0.016 (3)
N8	0.079 (4)	0.088 (4)	0.074 (4)	−0.040 (3)	0.007 (3)	−0.024 (3)
C29	0.053 (3)	0.074 (4)	0.044 (3)	−0.009 (3)	−0.011 (2)	0.007 (3)
C28	0.058 (3)	0.061 (4)	0.057 (3)	0.001 (3)	−0.012 (3)	0.017 (3)
N7	0.111 (5)	0.046 (3)	0.095 (4)	−0.017 (3)	0.032 (4)	−0.019 (3)
N9	0.059 (3)	0.058 (3)	0.056 (3)	0.000 (2)	−0.007 (2)	−0.017 (2)
C33	0.048 (3)	0.048 (3)	0.049 (3)	−0.009 (2)	0.003 (2)	−0.013 (2)
N10	0.095 (4)	0.061 (3)	0.073 (4)	−0.008 (3)	−0.031 (3)	−0.006 (3)
C34	0.063 (3)	0.043 (3)	0.056 (3)	−0.010 (3)	−0.013 (3)	−0.008 (2)

### Geometric parameters (Å, º)

**Table:**

Au1—Au2	3.1017 (3)	C11—C12	1.381 (7)
Au1—C32	1.983 (7)	C24—H24	0.9300
Au1—C31	1.987 (7)	C24—C23	1.388 (7)
Au2—C33	1.989 (5)	C24—C25	1.391 (7)
Au2—C34	1.994 (5)	C23—H23	0.9300
Ni1—N4	2.119 (4)	C15—H15	0.9300
Ni1—N5	1.994 (4)	C15—C14	1.393 (7)
Ni1—N6	2.110 (4)	C1—H1	0.9300
Ni1—N3	2.124 (4)	C1—C2	1.392 (8)
Ni1—N2	1.993 (4)	C10—C9	1.393 (7)
Ni1—N1	2.112 (4)	C18—H18	0.9300
N4—C20	1.346 (5)	C18—C17	1.375 (7)
N4—C16	1.344 (6)	C6—C7	1.399 (7)
N5—C21	1.351 (6)	C17—H17	0.9300
N5—C25	1.346 (5)	C27—H27	0.9300
N6—C26	1.353 (6)	C27—C28	1.368 (8)
N6—C30	1.344 (6)	C9—H9	0.9300
N3—C11	1.361 (6)	C9—C8	1.382 (8)
N3—C15	1.331 (6)	C14—H14	0.9300
N2—C10	1.352 (6)	C14—C13	1.370 (8)
N2—C6	1.343 (6)	C4—H4	0.9300
N1—C5	1.365 (6)	C4—C3	1.395 (9)
N1—C1	1.331 (6)	C12—H12	0.9300
C21—C20	1.481 (6)	C12—C13	1.381 (8)
C21—C22	1.379 (6)	C13—H13	0.9300
C20—C19	1.385 (6)	C7—H7	0.9300
C26—C25	1.493 (6)	C7—C8	1.373 (9)
C26—C27	1.391 (6)	C3—H3	0.9300
C16—H16	0.9300	C3—C2	1.338 (9)
C16—C17	1.380 (7)	C32—N8	1.146 (8)
C19—H19	0.9300	C8—H8	0.9300
C19—C18	1.381 (7)	C2—H2	0.9300
C22—H22	0.9300	C31—N7	1.150 (8)
C22—C23	1.380 (7)	C29—H29	0.9300
C30—H30	0.9300	C29—C28	1.381 (8)
C30—C29	1.396 (7)	C28—H28	0.9300
C5—C6	1.472 (7)	N9—C33	1.141 (7)
C5—C4	1.382 (7)	N10—C34	1.132 (7)
C11—C10	1.487 (7)		
			
C32—Au1—Au2	87.27 (15)	C23—C24—C25	117.8 (4)
C32—Au1—C31	179.0 (2)	C25—C24—H24	121.1
C31—Au1—Au2	93.69 (15)	C22—C23—C24	120.5 (4)
C33—Au2—Au1	94.33 (14)	C22—C23—H23	119.8
C33—Au2—C34	178.2 (2)	C24—C23—H23	119.8
C34—Au2—Au1	87.35 (15)	N3—C15—H15	118.5
N4—Ni1—N3	93.99 (14)	N3—C15—C14	122.9 (5)
N5—Ni1—N4	77.69 (14)	C14—C15—H15	118.5
N5—Ni1—N6	78.00 (14)	N5—C25—C26	112.9 (4)
N5—Ni1—N3	96.88 (15)	N5—C25—C24	121.3 (4)
N5—Ni1—N1	106.69 (15)	C24—C25—C26	125.7 (4)
N6—Ni1—N4	155.47 (14)	N1—C1—H1	118.9
N6—Ni1—N3	92.12 (14)	N1—C1—C2	122.2 (6)
N6—Ni1—N1	93.45 (14)	C2—C1—H1	118.9
N2—Ni1—N4	99.88 (14)	N2—C10—C11	114.0 (4)
N2—Ni1—N5	174.57 (15)	N2—C10—C9	120.2 (5)
N2—Ni1—N6	104.62 (14)	C9—C10—C11	125.8 (5)
N2—Ni1—N3	78.36 (15)	C19—C18—H18	120.1
N2—Ni1—N1	78.06 (16)	C17—C18—C19	119.7 (4)
N1—Ni1—N4	90.39 (14)	C17—C18—H18	120.1
N1—Ni1—N3	156.42 (15)	N2—C6—C5	113.6 (4)
C20—N4—Ni1	114.3 (3)	N2—C6—C7	120.6 (5)
C16—N4—Ni1	127.2 (3)	C7—C6—C5	125.8 (5)
C16—N4—C20	118.5 (4)	C16—C17—H17	120.8
C21—N5—Ni1	118.6 (3)	C18—C17—C16	118.5 (4)
C25—N5—Ni1	119.4 (3)	C18—C17—H17	120.8
C25—N5—C21	120.6 (4)	C26—C27—H27	120.4
C26—N6—Ni1	114.5 (3)	C28—C27—C26	119.2 (5)
C30—N6—Ni1	126.9 (3)	C28—C27—H27	120.4
C30—N6—C26	118.5 (4)	C10—C9—H9	120.9
C11—N3—Ni1	113.9 (3)	C8—C9—C10	118.3 (5)
C15—N3—Ni1	127.4 (3)	C8—C9—H9	120.9
C15—N3—C11	118.6 (4)	C15—C14—H14	121.0
C10—N2—Ni1	119.1 (3)	C13—C14—C15	118.0 (5)
C6—N2—Ni1	119.5 (3)	C13—C14—H14	121.0
C6—N2—C10	121.4 (4)	C5—C4—H4	120.4
C5—N1—Ni1	113.7 (3)	C5—C4—C3	119.1 (6)
C1—N1—Ni1	127.0 (4)	C3—C4—H4	120.4
C1—N1—C5	119.2 (4)	C11—C12—H12	120.4
N5—C21—C20	113.3 (4)	C11—C12—C13	119.2 (5)
N5—C21—C22	120.5 (4)	C13—C12—H12	120.4
C22—C21—C20	126.2 (4)	C14—C13—C12	120.1 (5)
N4—C20—C21	114.7 (4)	C14—C13—H13	120.0
N4—C20—C19	121.8 (4)	C12—C13—H13	120.0
C19—C20—C21	123.5 (4)	C6—C7—H7	120.9
N6—C26—C25	114.4 (4)	C8—C7—C6	118.2 (5)
N6—C26—C27	121.9 (4)	C8—C7—H7	120.9
C27—C26—C25	123.7 (4)	C4—C3—H3	119.9
N4—C16—H16	118.7	C2—C3—C4	120.1 (5)
N4—C16—C17	122.6 (4)	C2—C3—H3	119.9
C17—C16—H16	118.7	N8—C32—Au1	178.4 (5)
C20—C19—H19	120.6	C9—C8—H8	119.3
C18—C19—C20	118.8 (4)	C7—C8—C9	121.4 (5)
C18—C19—H19	120.6	C7—C8—H8	119.3
C21—C22—H22	120.4	C1—C2—H2	120.5
C21—C22—C23	119.3 (4)	C3—C2—C1	119.0 (6)
C23—C22—H22	120.4	C3—C2—H2	120.5
N6—C30—H30	119.0	N7—C31—Au1	179.0 (6)
N6—C30—C29	122.0 (5)	C30—C29—H29	120.6
C29—C30—H30	119.0	C28—C29—C30	118.8 (5)
N1—C5—C6	115.0 (4)	C28—C29—H29	120.6
N1—C5—C4	120.3 (5)	C27—C28—C29	119.6 (5)
C4—C5—C6	124.7 (5)	C27—C28—H28	120.2
N3—C11—C10	114.6 (4)	C29—C28—H28	120.2
N3—C11—C12	121.2 (5)	N9—C33—Au2	178.4 (5)
C12—C11—C10	124.2 (5)	N10—C34—Au2	178.9 (5)
C23—C24—H24	121.1		

### Hydrogen-bond geometry (Å, º)

**Table:**

*D*—H···*A*	*D*—H	H···*A*	*D*···*A*	*D*—H···*A*
C1—H1···N10^i^	0.93	2.57	3.235 (7)	129
C7—H7···N8^ii^	0.93	2.51	3.356 (9)	151
C8—H8···N9^iii^	0.93	2.54	3.421 (7)	157
C22—H22···N10^iv^	0.93	2.43	3.364 (8)	179
C23—H23···N7	0.93	2.51	3.274 (7)	139
C30—H30···N9^v^	0.93	2.41	3.280 (7)	156

Symmetry codes: (i) *x*−1, *y*, *z*; (ii) *x*−1, *y*−1, *z*; (iii) *x*, *y*−1, *z*; (iv) −*x*+1, −*y*+1, −*z*+1; (v) −*x*, −*y*+1, −*z*.
